# QR codes during the pandemic: Seamful quotidian placemaking

**DOI:** 10.1177/13548565231160623

**Published:** 2023-04-27

**Authors:** Hugh Davies, Larissa Hjorth, Mark Andrejevic, Ingrid Richardson, Ruth DeSouza

**Affiliations:** 5376 School of Media and Communication | Design and Social Context, RMIT University, Melbourne, VIC, Australia; 5376 School of Media and Communication, RMIT University, Melbourne, VIC, Australia; 2541 Communications and Media Studies, Monash Data Futures Institute, Monash University, Caulfield East, VIC, Australia; 5376 School of Media and Communication, RMIT University, Melbourne, VIC, Australia; 5376 Design and Social Context | School of Art, RMIT University, Melbourne, VIC, Australia

**Keywords:** Australia, digital practice, mobile media, pandemic, placemaking, QR codes, seamful, seamless

## Abstract

During the COVID-19 pandemic, one technology for contact tracing has come to dominate – QR codes. As a technology pioneered in Japan two decades ago and mainstreamed in China, QR codes have quickly become part of quotidian placemaking. While locations such as China have fully incorporated QR code technology into everyday contexts including public transport and mobile wallet applications, QR codes in the West were relatively overlooked. That was, until the pandemic. In this article, we examine some of the ways QR codes are being imagined and reimagined as part of public placemaking practices. In order to do so, we begin with a short history of QR codes – emerging in Japan, becoming mainstream in China and their consequent uptake globally. We then discuss the methods of our Australian study conducted during the pandemic and the seamful/seamless findings from our study.

## Introduction

This article explores some of the emerging perceptions and practices around QR codes as part of the COVID-19 response and its aftermath in Australian public spaces. While there has been much important work into mobile technologies as playing a crucial role in the curation of placemaking and how data is seamfully or seamlessly deployed in creative, citizen-orientated ways to reinvent spaces and places (Dodge et al., 2011; Frith and Ritcher, 2021; Farman, 2011; Nold, 2009), the implication of technologies to surveil has taken on greater significance during the pandemic (Andrejevic et al., 2021).We consider: does the constant physical checking-in – that is, seamfulness – make people feel more aware of giving away their data and being watched? Does it make them feel more in control? Does this awareness make for different movement patterns and awareness of data in public spaces? Or does it contribute to the use of digital media for sensemaking with others, data and places – what Halegoua and Polson (2021) call digital placemaking?

As COVID-19 spread around the world in early 2020, national governments began deploying a range of emergency responses. The rapid development of contact tracing apps featured prominently in many state and local solutions. In Australia, citizens were offered two contrasting technological solutions for addressing the challenge of contact tracing. The first was a Bluetooth app that operated passively, in the background, collecting information about which other phones a user had been in proximity with. App users only had to install and activate the app – it would do the rest. The second was a state-level QR code check-in app. This required the user to scan a QR code every time they entered a new controlled space – such as a shop or a café. Should someone who checked-in be diagnosed with COVID, those who had shared the space at the same time as the contagious person would be notified. Made compulsory in 2021, this system resulted in millions of check-ins each week, leading one privacy researcher in Australia to describe the check-in system as, ‘probably the most extensive surveillance operation Australia has ever seen’ (Greenleaf and Kemp, 2021). These check-in systems introduced radical new conceptions and experiences of space and place.

A key difference between the two apps concerns the extent to which they required a user’s conscious activity. If the government’s COVIDSafe Bluetooth app, once activated, operated ‘seamlessly’ in the background, QR codes were by contrast more ‘seamful’, in the sense that they required the ongoing check-in activity of users, who had to pull out their phones, activate their cameras, scan the codes and, in some instances, enter their information. In this respect, these technologies rehearse a broader set of issues associated with what Halegoua and Polson (2021) call digital placemaking. Digital placemaking describes the use of digital media to create a sense of place for oneself and/or others. As Halegoua and Polson (2021) argue, ‘the concept acknowledges that, at its core, a drive to create and control a sense of place is understood as primary to how social actors identify with each other and express their identities and how communities organize to build more meaningful and connected spaces’ (p. 1). These practices are deeply paradoxical – they empower as they exploit (Halegoua and Polson, 2021).

The acceleration of tracking technologies through the COVID pandemic has brought the implications of these practices in to sharp focus. For some time, we have found ourselves navigating the proliferation of usernames and logins in online environments that provide access to a growing range of digital enclosures (Andrejevic, 2007). One way to heighten data safety rights and awareness is through how *seamful* or *seamless* an application is. Drawing on a case study of the use of QR codes during the pandemic, this article considers the dynamic between ‘seamful’ and ‘seamless systems’ for access and control. Notions of seamlessness, which hark back to the important work of Weiser (1991) and the rise of ubiquitous computing, figure in more recent portrayals of digital and augmented reality. While initial aims/ideals centred around seamlessness application especially around mobile technology, contested practices such as seamful interfaces emerged (Bell and Dourish, 2014). Although early inventions and ‘killer apps’ of mobile technologies focused on ‘seamless’ convergence (Cumiskey and Hjorth, 2013) such as Bluetooth, technologies like QR codes re-emphasise the seam and suture – that is, the seamful. QR codes heighten awareness around data/information sharing and placemaking through their seamfulness (Hoffman et al., 2020).

The engagement with QR technologies has been uneven – emblematic of its divergent generational and cultural uptake across various COVID-related mobile apps across national and State systems. In popular media, humorous stories of misuse and seamfulness have proliferated. In New Zealand, for example, a young woman reported that her grandfather had taken multiple photographs of QR codes rather than clicking on the activated link (Wilkie, 2021). These stories highlight the diverse media literacies at play – mobile media often demand unspoken skills around data literacy, safety and awareness.

As we will reveal through our fieldwork, age, gender, ethnicity and profession all inform the variable and intersecting digital literacies and awareness that have emerged around contact tracing. Drawing from interviews in Australia, we argue that the implementation of QR code contact tracing has become a key barometer for understanding quotidian seamful mobile media publics. The passage through digital enclosure reconfigures what French philosopher Michel de Certeau (1984) described as ‘the arts’ of constructing everyday life as well as the relationship between overarching strategies of control and individual tactics of resistance. During the first pandemic of the ‘smart phone’ era, the range of technological responses reminded us just how thorough monitoring could be – and highlighted a range of possible responses.

In this article, we examine some of the ways QR codes have been imagined and reimagined as part of public placemaking practices. As we will see through our participants’ voices, some find QR codes a playful way to reintervene into everyday life and make moments more gameful, while others find it a tool for governmental public health control. We situate these responses against the background of a short history of QR codes – emerging in Japan, becoming mainstream in China and eventually taken up globally. We then discuss the methods of our Australian study conducted during the pandemic and the seamful/seamless findings from our study.

## Quick Read (QR) codes: A seamful history

Conceived by Denso Wave group in 1994, QR codes were developed to increase the information storage on a label as well as to speed up the scanning process (Denso Wave, 2019).^1^ The invention of QR codes was largely driven by a desire to improve one-dimensional barcodes (invented in US more than two decades earlier). While one-dimensional barcodes (still commonly used for products) are limited to around 20 alphanumeric characters and can only be read horizontally, QR codes allow for information to be presented and read both vertically and horizontally, while also holding 350 times as much information as standard one-dimensional barcodes (Denso Wave, 2019). In the face of stiff competition from other code scanning alternatives, Denso Wave decided not to exercise the legal rights of their patent, meaning that QR codes could be freely used by anyone, giving the codes a competitive advantage over alternatives (Karia et al., 2019).

While QR codes were initially developed for industrial applications, the rapid ubiquity of smartphones has effectively meant that everyone now carries a barcode reader in their pocket (Várallyai, 2013). The versatility and public accessibility of QR codes has seen them applied in a variety of settings to enable Offline to Online (O2O) transactions such as marketing campaigns (Asare and Asare, 2015), museum tours (Schultz, 2013), placemaking apps (Stokes et al., 2017) and location-based games (Koutromanos and Styliaras, 2015). While they have enjoyed some success in the West, QR codes have become an all-pervasive aspect of the Chinese urban landscape where they have been embedded into standard payment platforms WePay and Alipay (Hvistendahl, 2012; Yuan, 2017). By contrast, Western tech media outlets have for years projected the obsolescence of QR codes in favour of alternate technologies such as NFC (near field communication) and RFID (radio-frequency identification) tags or near field communication technology (Kaiserauer, 2017; Ochman, 2013; Pozin, 2012).

In previous scholarship, the use of QR codes for tracking has been proven as a robust, time and cost-efficient method in a range of settings including employee sign-in (Kumar and Kareemulla, 2017), tourism (Emek, 2012), ticketing, (Finžgar and Trebar, 2011) hospital in-patients (Fischer et al., 2013) and student attendance (Almasalha et al., 2014; Baban, 2014; Deugo, 2015; Koh et al., 2017; Talip and Zulkifli, 2018). At the heart of QR codes—as part of centralised tracing technologies – are issues around various forms of surveillance (governmental, corporate), privacy and security (Andrejevic et al., 2021; Lazar in Purtill, 2020). This debate enacts much of the discussion in Internet Studies around different forms of horizontal and vertical surveillance and their capacity for both benevolence and malevolence (Humphreys, 2011; Marwick, 2012; Zuboff, 2019). Not only have issues been emphasised around the capacity of governments to safely track and store information, but the possibility of ‘mission creep’ has also surfaced – that governments might extend the use of contact tracing into long term surveillance programs that continue after the pandemic has subsided (Busvine, 2020). As Morley et al. (2020) suggest, even the temporary or justified collection of sensitive personal data has ramifications for privacy, equality, fairness and inclusion. In the United Kingdom, for example, one in five adults does not use a smartphone, therefore some people may be excluded from contact tracing in terms of their access to technology or digital literacy.

Core to data rights and responsibility for users is how seamful or seamless an app is. Is the user constantly reminded of what they are giving away? In an incisive study exploring the ethical dimensions of contract tracing technologies in the Netherlands, Hoffman et al. (2020) note that the media coverage and scholarship concerning contact tracing apps has tended to fall along two lines: first, examinations of the intentions, efficacy, and utility of apps towards their stated goals; and second, the potential threats that apps and the data they collect might pose to the privacy and autonomy of individuals. Hoffman et al. (2020) investigate ‘seamless’ and ‘seamful’ approaches to contact tracing app design in order to uncover the affordances and values embedded into contact tracing apps, as well as the normative actions that design decisions might provoke among users.

Hoffman et al. make the case that more ‘seamless’ tracking methods such as Bluetooth ‘actively aim’ to bypass both individual user awareness and broader ethical frameworks. These technologies are very much part of early narratives around mobile media innovation as ‘seamless’. And yet, in this ideal of seamless convergence, mobile media practices constantly challenge this agenda – highlighting deep paradoxes whereby mobilities heighten immobilities (Cumiskey and Hjorth, 2013). As Cumiskey and Hjorth note:

the smartphone is now ushering in new promises of seamlessness between engagement with technology and everyday common experiences. This seamlessness is not only about how one transitions between the worlds of the device and the physical environment but it also captures the transition and convergences between devices as well (i.e., laptop to smartphone, smartphone to tablet)… however, these transitions are far from seamless. We see divisions between online and offline, virtual and actual, here and there, taking on different cartographies, emergent forms of seams (2013, p. 1).

Hoffman et al. suggest that more seamful methodologies such as QR codes make users more explicitly aware of the ‘suture’ involved in the transfer of personal information to centralised databases – thus encouraging enacting responsibilisation. In the Australian context, the Federal Government’s centralised COVIDSafe app uses Bluetooth to detect the proximity of other individuals via their devices. In contrast, scanning QR codes requires individuals to manually perform familiar and habitual embodied actions, a series of haptic screen gestures that concludes with a deliberate tap on the “Check In” button. This shift from *seamless* to *seamful* interaction affects the way users perceive and experience contact tracing and their agency in this process. So how does the seamful nature of QR codes shape people’s experiences of moving through public spaces in relation to data and tracking? In the next section, we discuss the adoption of QR codes in Australia during the pandemic.

## QR codes in Australia

With Australia’s COVIDSafe contact tracing app beset by privacy concerns (Leins et al., 2020; Thomas et al., 2020), technical problems (Taylor, 2020; Thomas et al., 2020) and perceptions of being an overall failure (Dev, 2020; Kearsley, 2020; ), QR codes have emerged as an ad-hoc, inexpensive, contactless and rapidly deployable check-in solution (Greenleaf, 2021; Purtill, 2020). In Australia, through November and December of 2020, QR code check-in methods became mandatory for hospitality venues and other indoor premises in ACT, NSW, WA and SA. Meanwhile, other States such as Victoria and Tasmania introduced QR code solutions across a range of venues. To use the QR code for contact tracing, a smartphone will ‘scan’ the code with the camera function or QR code reader app. The user will then be directed to a website or mobile app to enter their details. Users download a government app and complete a short online form, the information from which will then auto-fill on all future scans. In 2020, prior to the launch of all official government QR code apps, some non-government custom QR code providers saw users needing to enter their personal details for each venue and scan. If users did not have their smartphone with them, they could record their details manually with pen and paper (Government, 2021).

The QR code system was inconsistent both across and within states. While most states developed their own QR code check in systems, these solutions did not align with a national approach (Hickey, 2021). Into this gap between state and national systems, a number of small provider companies rushed into the QR code tracing space. Some of these check-in apps were owned by companies that deal in data collection and whose processes for how that information was stored and used was not transparent (Purtill, 2020). Multiple concerns have been raised – data can potentially be hacked or simply sold, used for identity fraud or to track a person’s location and social networks, or to micro-target advertising or misinformation campaigns (Nguyen, 2020). The Australian Government Cyber Security Centre warns that it is ‘quick and easy’ for anyone to generate QR codes for a range of intentions, including to obtain personal information, to cause a smartphone to visit a harmful website, install malicious software, or join an untrustworthy network (Australian Cyber Security Centre, 2021).

Despite the potential privacy concerns associated with inconsistent rules across different QR code systems deployed in different locations, the response to the system rollout was relatively muted compared with the attention received by the national contact tracing COVIDSafe app. Here, the convenience of using QR codes to access sites clearly outweighs the fears of surveillance – highlighting Andrejevic’s earlier work on perceptions of, and sensemaking with, the sharing of personal information (2013). As he notes in *Infoglut,* Australian participants interviewed often gave away their data to corporations for the convenience of services such as WIFI (Andrejevic, 2013). During the pandemic, notions of data sharing for public health or ‘social good’ have permeated State government messaging. And so, how does the social good of data sharing, the seamful nature of QR codes in tracing and tracking configure the choreography of digital placemaking practices in public spaces. In the next section, we explore some of the perceptions and practices around QR codes and contact tracing from fieldwork conducted in Australia.

## Methodology and context

Commencing in early 2020, the *COVID: Australian practices and perceptions* project has evolved over a 2-year period of the pandemic, becoming a longitudinal study involving participants from across Australia. The interdisciplinary team had expertise in nursing, media and communication studies (specialising in mobile media practice), cultural safety, socio-cultural practices of technology and urban space methodologies. Data was primarily collected through surveys and interviews.

The project evolved over three phases of surveys followed by interviews. Each phase sought to capture the different perceptions (survey) and practices (interviews). The iterative project involved various phases that responded to the different contingencies of the pandemic. It was important to use different methods such as surveys and interviews to address the various attitudes, perceptions and practices during the pandemic. While surveys address perceptions and tastes, interviews provide the ability to do deep dives into understanding media practice – that is, motivations for use. Interviews focus on the why and how rather than the what (surveys). We also deployed follow-up interviews (taken 6 months after the first interviews) to identify dynamics and changes during the pandemic as fatigue and exhaustion from endless lockdowns ensured.

Beginning with the COVIDSafe App survey *(Phase 1)*, we then interviewed participants twice over a 6-month period *(Phase 2)*, followed by a frontline workers’ survey and interviews *(Phase 3)*. In this paper, we focus on the interviews conducted in Phase 1 and 2 in which different mobile technologies for contact tracing were deployed. Phase 1 was conducted from May to September 2020. It consisted of a survey of 130 participants, with follow-up in-depth interviews of 20 participants between the age of 20 and 65 selected to include a range of locations, professions, and backgrounds.

During this period in which the interviews took place, lockdowns occurred in most major cities and a state of emergency was declared in Victoria, with the state going through 6 months of restrictions that included curfews from 8 p.m. to 5 a.m. and 5 km travel limitations. The interviews conducted during this period sought to explore pro-social practices of self-surveillance and surveillance of others, by asking people to reflect on their own attitudes, behaviours and perceived responsibilities, their perceptions and use of mobile apps, contact tracing and media technologies, and their observation and ‘policing’ of the practices of family members, friends and ‘familiar strangers’ in public spaces. We explored the uneven rollout of contact tracing systems in which State governments trialled various mechanisms.

Six months after the initial research collection, we added an additional phase and undertook follow-up interviews in April and May of 2021. In the second round of interviews, we spoke with 10 of the original participants to explore how some of their perceptions and practices had evolved over time. During this phase of the research, interviews were undertaken via video chat or over the phone due to lockdown restrictions. A set of guiding questions shaped the interviews and provided an opportunity for open conversations of approximately half an hour each. We sought to discover the personal and social contexts framing participants’ perspectives, and upon analysis several themes were identified. All participant names used are pseudonyms. The interviews were semi-structured around questions relating to technology, public space, data and pro-social activities (mask wearing and social distancing). The interviews were structured to ensure that participants were recognised as experts – that is, experts of their own lived experience.

For this paper, we focus on eight participants who were re-interviewed 6 months later to explore some of the diverse experiences across age, gender, profession and cultural differences and the role of QR codes for quotidian ‘seamful’ mobile media. The role of seamfulness becomes a site for everyday citizens to take back control in their digital placemaking practices. We reflect upon how they felt about these practices – did the constant checking in make them more mindful about data, datafication and dataveillance? Did they think of this as empowering or exploitative? Did the datafication of their movements make them more conscious and deliberate and even change practices?

## Contact tracing in context: Australian perceptions

These seamful and seamless technologies enact an inversion of de Certeau’s famous ruminations about urban pedestrians, ‘whose bodies follow the thicks and thins of an urban “text” they write without being able to read it’ (1984, p. 128). The QR code signposts come to serve as textual markers – checkpoints along the way that promise the newfound legibility of the urban (and suburban) text. At the level of the individual user, these checkpoints retain a degree of inscrutability: What are they doing? Did they work? Where did that information collected from my phone go? How does this knowledge impact upon our understandings of place and placemaking?

These QR checkpoints also cultivate an imagined position from which the texts they are tracing can be reconstructed in productive and protective ways. de Certeau describes this perspective as the view from on high which tempted Icarus: ‘It transforms the bewitching world by which one was ‘possessed’ into a text that lies before one’s eyes. It. allows one to read it, to be a solar Eye, looking down like a god’ (1984, p. 127). Sunlight’s disinfecting power takes on renewed salience in the face of the viral threat. The perspective from ‘on high’ promised to both illuminate and contain contagion.

Did QR codes make participants feel more empowered as they moved through public spaces and ‘texts’ as de Certeau would say? Or do QR codes enact the difference between de Certeau’s (1984) notion of tactics and strategies? For de Certeau, everyday life was a constant contestation between strategies and tactics. Strategies are mechanisms deployed by the government and state to control the subjugated, whereas tactics are mechanisms deployed by the repressed for creative subversion. The collection of data in everyday life is omnipresent – dubbed datafication. Our mobiles are constantly leaking information about us to corporations and governments. And yet, in a de Certeau sense, they are not just strategic vehicles for repression. Rather, it is through seamful technologies that citizens are made aware of the datafication and can find expressive and creative ways for placemaking. Indeed, QR codes allow us to think through the possibilities and limitations, strategies, and tactics of quotidian placemaking practices.

In our surveys and interviews, participants expressed different feelings and emotions towards the techniques of contact tracing. Some found QR codes expressive and playful, others found it a reminder of the datafication of everyday life. Dimensions such as age, gender, cultural, and linguistic differences informed how the various contact tracing mechanisms were viewed. As aforementioned, we conducted two sets of interviews 6 months apart to understand some of the dynamic responses in attitudes, perceptions and practices around the changing technologies for contact tracing. In this section, we focus on eight diverse participants to provide insights into the ways in which QR codes as seamful devices are read across gender, age, profession, and cultural differences.

We begin with Chloe – a white woman in her twenties living in Western Sydney. She thinks the COVIDSafe app was a failure and has only heard jokes about it. For Chloe, the various forms of contact tracing and the attendant collection of personal information makes her nervous of manual pen and paper check in, Chloe remarks: ‘You see everyone … that came in before, you see their information as well. So, there’s like … private information just leaking everywhere’. Here, the seamlessness is viewed as a problem by which we leave constant data trails as we move through public spaces.

Chloe felt that QR codes offered the perfect solution for contact tracing; much better than leaving details or the COVIDSafe app. It made people more mindful of (‘seamful’) tracing rather than the COVIDsafe app which seamlessly used Bluetooth in the background. However, Chloe was concerned about how that data will be used later. ‘I’ll put that app on my phone if you want, but what’s to stop that from staying there or these tracing measures staying here after COVID has gone? That’s what I’m worried about’.

Gender clearly informed participants’ responses to the different tracing systems. In our surveys and interviews, female participants raised more concern towards surveillance or privacy issues – discerning in the seamful nature of QR codes the potential for detriment in how data can be collected and redeployed. There was, in these responses, a sensitivity to the tension between the data strategies and tactics of everyday life – between possible forms of shaping and controlling data collection on the one hand, and concerns about its alignment with unseen powers for undisclosed purposes.

White, Melbourne-based software development consultant Mike in his late 40s, describes himself as ‘reasonably privacy sensitive’. He thought the COVIDsafe app was a good idea, but admits he is already ‘well-tracked’ by corporations such as Apple and Google. Mike is overtly aware of data strategies in a de Certeau sense, but less concerned about what it means for him. He decided—in keeping with Andrejevic’s (2013) findings that many will surrender privacy for convenience – ‘well if I’m prepared to give up that information for just a little bit of convenience, I should be prepared to give up the same information in order to potentially save lives’.

In a follow-up interview with Mike 6 months later (April 2021), when QR codes tracing was being made mandatory, he spoke about the inconsistent implementation of contact tracing processes. Prior to the implementation, Mike had very little access to QR codes, despite working in the IT industry – highlighting the fact that QR codes have gone from relative obscurity to mainstream contact tracing use during the pandemic: ‘I’d used them (QR codes) a tiny bit but very little. I’d say normally to just use them occasionally’.

Mike’s experience described the shift of QR codes from a relative novelty to something people had to accustom themselves to using on a daily basis. A visit to Adelaide in South Australia gave him a look at what a centralised tracking system looked like (since, for a time, Melbourne used a patchwork of different QR code-based systems). When the State of Victoria (where Melbourne is located) deployed its own, centralised app, it was, as Mike noted, not without some potentially confusing elements: the new system required some extra steps, including downloading a dedicated app and thus changing the way check-in worked.

As someone who is highly media literate and specialises in technology, Mike said he had no concern about giving over his details for the ‘social good’ of contact tracing. However, he felt some systems lent themselves to being compromised – such as information being passed on to marketers. He also felt Victoria had been “slack” not deploying a mandatory QR code system from the beginning like SA. For Mike the seamfulness of QR codes is not just in their operation on phones across the official State app and camera app but also across the different State systems. While SA adopted one system deployed through the State App, Victoria adopted a hybrid approach which was, over time, streamlined into the State App, *Service Victoria*.

Jordan, a white male in his early 40s working in the creative industries and experienced in tech development, admitted that he doesn’t always check-in. ‘If I’m just ducking in, like if I’m going to the supermarket for one thing, I generally won’t bother’. However, Jordan isn’t too concerned about contact tracing with QR codes. He thinks tracking has largely become normalised even pre-COVID. It’s just the ‘mild inconvenience’ of it in which an extra layer is added onto the datafication of everyday life. For Jordan, QR codes remind him of games like Foursquare in which placemaking is gamified:

If anything… I think about it (QR codes) more in terms of *Foursquare,* the old location-based game app. You would check into locations and the more you checked, the more kind of badges and points you could earn.

Here Jordan identifies the seamful role of QR codes as making users conscious of their location and others. Jordan identified that QR codes during the pandemic are a form of gamifying how we move through public spaces and placemaking. The overlaying of data, place and movement allow for ways in which to be more conscious, and thus more playful, with sensemaking around places. Jordan reports that the QR code check-ins remind him of locative games and thus represent playful placemaking for him. Much of the pandemic visualisations of statistics seem to gamify life and death in ways he sees borrows from game-like techniques like leaderboards and comparative graphs. Jordan confesses to an excitement in discovering a ‘near misses’ as he moves through the city and the latest reported hotspots. He also admits that ‘there’s some addictive nature to obsessively checking’.

This playful or tactical interpretation of QR codes in digital placemaking was shaped by gender with many of our female participants expressing more concern around privacy trade-off for the social good. Cultural and age distinctions were highlighted in interviews with two Chinese participants Shirley and Jasmin. Shirley, a Melbourne woman from Hong Kong in her 60s, had more difficulties logging in and using the QR code apps than Jasmine who found the system mundane and easy.

Although initially concerned about the outbreak, Chinese medicine mature-aged student Shirley was sceptical of much of the common thinking around COVID-19 restrictions and contagion. Her school used QR code scanning, but she found the process difficult and complicated (it required her student email account and password to complete the process). She was not especially concerned about the tracking of her information as broadly she trusts the Australian government. When we followed up with Shirley several months later, she had become more accustomed to QR code contact tracing, but she expressed some reservations about it. Specifically, she was concerned about her movements being tracked – not by the government, but by how this information might be exploited by other businesses or organisations beyond the COVID crisis.

Some similar concerns about exploitation of data arose for Jasmin, a Melbourne-based reporter and academic in her 20s from mainland China. While she initially downloaded the COVIDSafe app, a few weeks later, when it was asking her for location permission, she became a bit concerned about her data privacy and deleted it. Jasmin found the QR code system easy to use with her iPhone and was familiar with it as a system used commonly in China and through mobile apps like WeChat. For Jasmin, QR codes were a mundane, trusted technology that facilitated mobile placemaking. Nonetheless, Jasmin, felt uncomfortable and exploited when some of the non-government QR code apps sent promotional information to her email. Jasmin told us: ‘I think they need to make the system more transparent and at least tell people, and tell people how the data would be stored or used, and for what purpose’.

Age was a factor in how systems were perceived as ‘easy’. Just as we saw with Shirley and Jasmin, divergences could be found when comparing two of our participants, Mike and David. Mike, like David, didn’t have concerns around privacy when weighed up with convenience. A semi-retired white man in his early 60s, David believed the COVIDsafe rollout had been managed quite well and that the initiative made ‘good sense’. He didn’t understand why people objected to or had concerns about privacy (they’re ‘a bit nutty’), and that he ‘almost felt the app should have been mandatory’. He felt the technology had been ‘politicised’ particularly by the ‘left side of politics’.

In our second interview with David in April 2021, the discussion had shifted from the COVIDSafe app to QR codes. As he noted, QR codes are ‘a piece of cake, it’s easy, you just take a picture with your camera and just put your details in. I find it’s completely easy, it’s simple.’ David had initially thought he needed to have a QR code reader open. Unlike Jasmin who had experience with using QR codes prior to the pandemic, David had only recently become familiar with them. David added that he found the COVIDSafe app easier – because it was seamless once it was turned on the user didn’t need to do anything – than the seamful QR codes that constantly reminded him he was moving and leaving a data trail. However, David’s responses also evidence a level of pandemic fatigue that he associated with the contact tracing. He tells us:

Somewhere underneath all the rational thought there’s a desire to just not have to worry about doing all this stuff. I don’t want to have to do QR codes for the rest of my life. I don’t want to have to have an app … I’d just rather get back to how things were.

There was also resistance to downloading the *Services Victoria* app (for QR code check-in) on the part of some of our respondents. The app connected to the government public health system (Medicare) and could store people’s vaccination certificate – something that became mandatory for entrance into many sites from around September 2021. Putri, a Melbournian woman in her 20 s who works at a local city council office, resisted installing the app because of privacy concerns – until she was required to use it when she returned to work at her office after lockdown restrictions eased: ‘I’m not big on downloading apps and then that gives access to my other information on the mobile’.

According to the State’s online information about the *Services Victoria* app, the data it collects is limited to the user’s name and phone number (entered by the user) as well as time, date, and location of entry (recorded automatically by the app). However, Putri is generally wary of downloading apps precisely because of their ability to collect information automatically, and she does not necessarily trust published claims about the limits on what information is being gathered and stored. In this respect, she identified one of the big perceived differences between the app and its analogue alternative – writing your name, phone number and time of arrival down on a clipboard. This alternative was necessary, since not everyone has a mobile phone, and not everyone brings one to every shop and venue they visit.

Like Putri, Kusuma, an international female graduate student from Singapore, avoided installing the *Services Victoria* app, preferring systems that link the code straight to a website. When the QR code links to a request to download an app – typically the State sponsored one – she declines: ‘I just stop at a point, and I don’t want to download another app onto my phone’. Both respondents noted the inconsistency with which the use of the app is enforced – perhaps a legacy of the piecemeal way in which the QR code system was deployed in Victoria. Kusuma noted seeing people skip the QR check in – and the lax enforcement on the part of venue managers that made this possible:

I find that people only do it when other people are doing it. So, like say if there is a load of people and they are all coming in through one entrance, and like if the first person scans the QR code, then everyone after that first person does it. But there are times when there are like very few people, or like, you know, just people trickling through the entrance, and then they will just walk in and they won’t even scan.

As our participant discussion has highlighted, relationships to QR codes and levels of trust, privacy and accessibility varied according to various factors. For some like Jordan, the playful/gamified nature of QR code check-in was reminiscent of locative apps like Foursquare which added a layer of tactics onto digital placemaking in the everyday. For David, the seamful nature of QR codes was a nuiscence. While for Jasmin it helped increase her everyday awareness of datafication. As we see through the perceptions of our eight diverse participants as a snapshot of our broader findings – age, gender, cultural background and profession all inform the divergent experiences and perceptions around QR codes and contact tracing. By mapping some of the perceptions and practices informing the implementation of contact tracing from COVIDSafe to QR codes, we can gain a sense of underlying relationships to media, privacy and social responsibility. As we have explored, QR codes for contact tracing are about seamful placemaking that heighten awareness around datafication, privacy and trust. Depending upon the participant, they were viewed as ‘strategies’ or ‘tactics’ in a de Certeau sense.

## Conclusion: QR codes as quotidian seamful placemaking

As we move globally yet unevenly in and out of the pandemic, by exploring contact tracing mechanisms – and the perceptions and practices that underscore them—we can learn a lot about the role of media for seamful placemaking and its relationship to cultural perceptions around social responsibility. As we have argued, the ‘seamful’ nature of QR codes create and curate a sense of mindfulness and awareness to data privacy – it constantly reminds users that they are checking in and thus sharing their data and placemaking movements with others. The seamfulness of QR codes makes participants more aware of the implications of datafication in everyday life. For some, this is about affording a sense of expression and tactics (Jordan), for others it reminds them of datafication strategies in which privacy and trust come into play (Jasmin). While all participants viewed QR codes as seamful – some viewed this as positive and productive (David and Mike), while others viewed it as indicative of datafication in which they have little control or agency (Putri and Kusuma). Increasingly, research around the use of mobile technologies in urban spaces by women and non-white participants reflects the need for greater understanding into safety and privacy. Just as witnessed during the *Pokémon Go* phenomenon, non-normalised bodies such as black men in public spaces were policed and killed while white, normalised bodies continued playing their game un-interpreted (Davies and Innocent, 2017; Hjorth and Richardson, 2017; Salen Tekinbaş, 2017). Unlike the seamlessness of applications such as AR in *Pokémon Go,* QR codes are a constant bricolage of material and digital overlays and actions, highlighting agency in seamfulness.

As we have suggested the seamful/seamless metaphor highlights the role of mobile technologies for de Certeau’s notion of *repressive s*trategies (organisational control over the subjugated) and *expressive* tactics (mechanisms deployed by the subjugated). While QR codes are seamful, in their conspicuous nature they also highlight the increasing role of mobile media in digital placemaking. They remind us of the constant leaking of information as articulated by out participants. They remind us that places are increasingly interwoven by seamful and seamless, mobile and immobile media practices that impact upon our sense of sensemaking in and with others. Moreover, they anticipate the further development of digital infrastructures associated with AR, which continue to negotiate the relationship between the seamful and seamless. Taken to its limit, virtual reality envisions the prospect of mediation disappearing into ubiquity. At the same time, these technologies also raise the somewhat paranoid prospect of the centralized orchestration of reality itself. AR plays with the seams rather than eliminating them, envisioning the prospect of moving back and forth between the physical and the virtual, the material and the informational. In this context, our respondents raise important issues that are likely to become more pressing as the technology develops.

As we have highlighted, these seamful and seamless practices have a double valence: on the one hand, we have considered, over the course of this article, the varying responses on the part of our respondents to a new array of semi-compulsory technological practices; on the other hand, however, the technology promises to render their movements increasingly legible for the purposes of securing public circulation while simultaneously containing viral spread. In the former case, the uptake of the technology is conditioned both by varying levels of comfort with respect to the interface and of trust regarding what takes place behind it. In the latter, the deployment of the QR code represents another halting step toward the implementation of widespread forms of interactive spatial monitoring. This phenomenon is shaped by the choreography of seamful and seamless practices.

The space-making practices associated with the use of the QR code represent a hedge against the threat of stasis imposed by the pandemic. We know, historically, that the legacy of the plague is enforced quarantine: the attempt to thwart the spread of the virus by rendering its carriers – us – immobile, sequestered in our homes. We also know that in the contemporary conjuncture, immobility spells economic devastation. The high turnover, just-in-time, transit-dependent economy we have created for ourselves has a very low tolerance for imposed forms of stasis that stall supply chains and slow consumption. There is a redoubled urgency, then, to cultivating the practices that enable secure circulation. Perhaps, for the first time in history, we have the technological infrastructure in place to allow us to imagine the prospect of granular monitoring of mobility – the creation of a centralised record of where everyone has been for the past 2 weeks (or more): to retrace their contacts and their contacts’ contacts in something close to real time.

## Note

1. Denso Wave is a subsidiary of the Toyota Company.
